# A Classification-Lock Tracking Strategy Allowing a Person-Following Robot to Operate in a Complicated Indoor Environment †

**DOI:** 10.3390/s18113903

**Published:** 2018-11-12

**Authors:** Shenlu Jiang, Wei Yao, Zhonghua Hong, Ling Li, Cheng Su, Tae-Yong Kuc

**Affiliations:** 1 College of Information and Communication Engineering, Sungkyunkwan University, Suwon 440-746, Korea; sljiang@skku.edu (S.J.); liling0528@skku.edu (L.L.); su920304@skku.edu (C.S.); tykuc@skku.edu (T.-Y.K.); 2 Department of Land Surveying and Geo-informatics, The Hongkong Polytechnic University, Hung Hom, Hong Kong, China; wei.hn.yao@polyu.edu.hk; 3 Research Institute for Sustainable Urban Development, The Hongkong Polytechnic University, Hung Hom, Hong Kong, China; 4 College of Information Technology, Shanghai Ocean University, Shanghai 201306, China

**Keywords:** human-following robot, person localization, robot application

## Abstract

Person-following technology is an important robot service. The major trend of person-following is to utilize computer vision technology to localize the target person, due to the wide view and rich information that is obtained from the real world through a camera. However, most existing approaches employ the detecting-by-tracking strategy, which suffers from low speed, accompanied with more complicated detecting models and unstable region of interest (ROI) outputs in unexpressed situations. In this paper, we propose a novel classification-lock strategy to localize the target person, which incorporates the visual tracking technology with object detection technology, to adapt the localization model to different environments online, and to keep a high frame-per-second (FPS) on the mobile platform. This person-following approach consists of three key parts. In the first step, a pairwise cluster tracker is employed to localize the person. A positive and negative classifier is then utilized to verify the tracker’s result and to update the tracking model. In addition, a detector pre-trained by a CPU-optimized convolutional neural network is used to further improve the result of tracking. In the experiment, our approach is compared with other state-of-art approaches by a Vojir tracking dataset, with three sequences in the items of human to prove the quality of person localization. Moreover, the common challenges during the following task are evaluated by several image sequences in a static scene, and a dynamic scene is used to evaluate the improvement from the classification-lock strategy. Finally, our approach is deployed on a mobile robot to test its performance on the function of the person-following. Compared with other state-of-art methods, our approach achieves the highest score (0.91 recall rate). In the static and dynamic scene, the output of the ROI based on the classification-lock strategy is significantly better than that without it. Our approach also succeeds in a long-term following task in an indoor multi-floor scenario.

## 1. Introduction

Person-following is an important robot service, and it can be employed in various scenarios, i.e., autonomous wheelchair-following for accompanying people [1], an item carrier in shopping malls [2], etc. Generally, the following robot will operate in a dynamic and complicated environment, in which it may meet several environmental challenges. For example, the target person may be covered or partly covered by other persons or obstacles, the illumination situation may suddenly be exposed or darkened by the influence of changing light sources, the target appearance may change during the task (e.g., the target person changes clothes, puts on a hat, or removes their bag, etc.) and the poses of the target may change (e.g., squatting down or picking up something from the floor). Therefore, how to improve the preciseness and robustness for person-localization is a key issue for the person-following function.

The major issue of person-following is how to localize the target person by tracking. Such methods monitor the localization of the target person over time and identify them based on motion continuity. One issue concerns sensor selection. In mobile robot platforms, range data, especially that determined by a laser scanner, are often used for person-detecting and -tracking, because of its accuracy and the wide view in range searching [3,4,5,6]. It detects the person based the shape of the human scanned by the range data, and tracks the person through the continuous movement of their position. However, signal loss happens when the person is occluded by others for several seconds, and the person-localization system of the robot may then track the person that occluded the target, because the shapes of humans as depicted by the representation of range data are similar. Therefore, accurate identification of the target person is essential for this work. Different from the state of range data, methods based on image data take advantages in identifying the target person, because image data contains visual image information from the real world. Because of the strength of significant features that are extracted from human appearance, the above methods can identify the correct target, overcoming the challenges of cover or partial cover. Similar to the accomplishment of range data, methods based on image data detect the target person in the scene based on feature points [7,8], skeletons [9,10], gestures [11], stereo information [12], or by combining target’s height, path, and the color of cloth [13], and identifies them by their continuous movement. However, there are two major drawbacks, i.e., limited adaption from model to the environment, and high computing cost. Detection with a higher accuracy generally brings with it more exhaustive computing costs, because of the greater complexity of the detection model, e.g., the method based on deep learning [14]. However, even though the deep learning approach can obtain magnificent accuracy and operate in real-time under high-performance GPU support, exhaustive computing costs restrict it from operating on a mobile platform (it can only operate on a CPU). Differing from traditional methods, the method based on deep learning learns features through the computer itself, and it overcomes limited representational strength, through human-designed features. However, one other issue arises, in that the training data always have undescribed poses or unexpected appearances of humans, which leads the ROI (region of interest) of the target to sharply flash or be lost; this causes a loss of the target’s localization. Such a situation will cause uncontrollable movement from the person-following robot, and lead to a failure of the following task.

**Figure 1:**
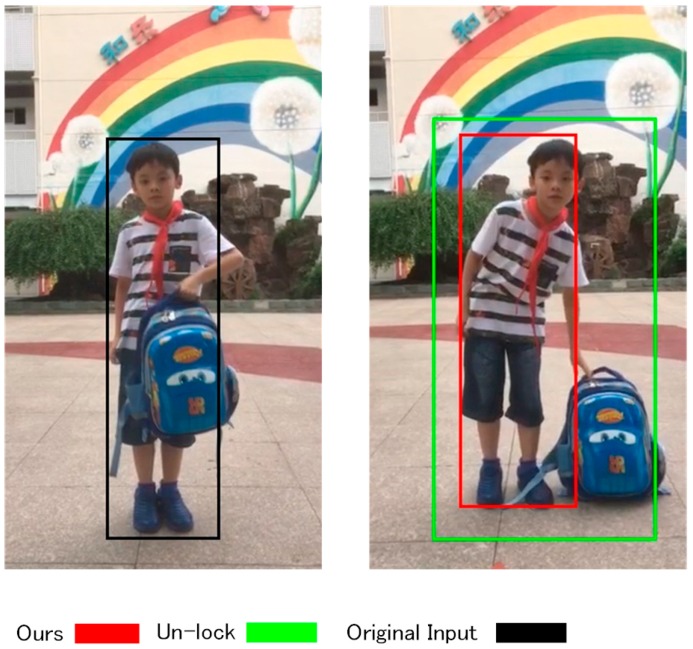
Person localization samples. The un-lock marker depicts the results without a classification-lock strategy, and the red marker depicts the classification-lock strategy.

In summary, ROI flash or loss, no matter whether the type of method used is based on range data or image data, is inevitable, because of the complicated background and the appearance of persons in the real-world environment. Simply extending the training dataset can somehow benefit the accuracy of detection, but unknown environments will always exist, which makes the ROI flash or loss still occur. To improve this issue, we propose the addition of an online feature learning procedure, in order to adapt our model to the environment during the following task. Our approach focuses on constantly localization one target person, and regarding all other objects (no matter human or background) as the background. Visual tracking technology (for a review, see [15]) can be used to play the role of an online detector to localize the target person. One trend is to use a tracking–learning–detecting [16,17] strategy, which incorporates the tracker with a detector. In tracking–learning–detecting strategies, the tracker follows the movement of the object, the detector justifies the tracker’s result between two frames, and the learner updates their models. One factor that should be noted is that the major difference between tracking–learning–detecting and detection-by-tracking is that the model localizes the target object, based on an online- or offline-trained model. One drawback that appears for the visual tracking is that the tracker only utilizes the online feature into a learning procedure, and the person-localization model is a lack of restriction on the object’s classification, shown in Figure 1; i.e., the online learner considers the feature-rich bag as a target, and it puts the intensity of tracking on the bag which leads the outlier happens. Apart from this, the tracking–learning–detecting technology also faces the challenges of scale, rotation, appearance change, etc. Our approach employs the idea of the tracking–learning–detecting strategy, which employs the center cluster approach [18] as a tracker, the PN classifier [19] as a detector, and an updating procedure as the learner. The [18] is a part-based tracker that splits the appearance of the objects into different parts, and follows each part through the tracking procedure. The edge of human appearance is significant in the image scene, which allows for good performance by the part-based tracker. However, the tracker is based on feature points, which mainly represent the extreme points in the image, but not the texture on the object. This issue occurs when two persons overlap; the tracker may lose the correct target because of a similar distribution of feature points. To improve this case, the PN classifier is selected to justify the tracker. The PN classifier is trained online by positive samples (target person’s appearance) and negative samples (background), which justifies the tracker according to the learnt texture feature of the target person and the background. The PN classifier selectively searches the region around the tracker’s output, and evaluates the most confident region of the target. 

One major difference is that we employ the CNNs detector to restrict the model, in order to focus on the human’s appearance, to serve as the initializing step. The neural network is trained by our own selected datasets. The CNNs detector is first employed to detect the human’s positions in the first frame, to give an initial position of the target to the person-localization model. After each step during the tracking procedure, the CNNs detector is used to justify the confident rate of the output for humans, in order to avoid the occurrence of the issue, as in Figure 1. There are several existing detectors that are based on a deep neural network. Starting from R-CNN [20], the two-stage approaches (finding the objects’ potential positions and detecting the high confident candidates), fast and faster R-CNN [21,22] obtain excellent detection quality, but the real-time processing is a challenge, even with GPU enhancement. Another trend is the one-stage approach, such as YOLO [23], in which researchers convert the detection task as a regression work, and it obtains a timeless detecting speed; however, the accuracy of YOLO is relatively lower than other state-of-art methods, and the training procedure is highly dependent on the parameters to be set. Moreover, SSD [24] combines the advantages of faster R-CNN and YOLO, which possess fast and accurate detection performances. For the purpose of deploying on the mobile platform, SSD and various optimizations are employed to improve the speed of processing, with limited detection of quality loss. To reduce the time during the processing of the convolutional layers, our approach utilizes the MobileNet [25] as a back net, and it employs the depthwise convolutional layer instead of the traditional convolutional layer. Moreover, the neural network is re-designed with tiny input resolution (128 × 128) to reduce the computing cost from large resolution neural networks. Because of the connection between two frames, the output through the tracker will not be a great outlier. Therefore, we employ the slightly extended region that is output by the tracker as an input of the neural network, in order to allow the neural network to rapidly localize the person.

In this paper, to prove the efficiency of our person-localization approach, we deploy various experiments to evaluate the common challenges on the person-following task, in both simulated and real-world operations. Firstly, our approach is evaluated both with and without a classification-lock strategy by the occlusion, re-detection of the appearance of similar people, scale change, rotation change, and the target’s appearance change, in four recorded image sequences. The results show that our approach can overcome the above challenges. The classification-lock strategy improves the ROI output, and assists our approach of avoiding the failures singly, by using detection or visual tracking. Thus, a real-world following task is operated. To allow for the movement of the robot, a simple PID controller, which keeps a fixed distance between the user and the robot, is employed to drive the robot. The result shows that our robot accomplishes the human-following mission against environmental influences, with a long-term following travel time, and it allows for the following service to operate among the floors. To prove its efficiency, our approach is compared with four state-of-art tracking methods on the Vojir tracking dataset [26] whose subject is humans. The result demonstrates that our approach obtains a state-of-art recall rate, and shows the success of our classification-lock strategy. 

Prior to this paper, we presented in [27] an original version of this work. The initial version incorporated the tracker, PN classifier and a Yolo based deep neural network to accomplish the classification-lock function, which successfully allowed a following robot constructed on an Intel Nuc platform to follow the user in a single floor. However, there were three major limitations: (1) The system can only reach 13 FPS on our platform; (2) The Yolo model required a dramatically long training time; (3) The experiment was operated in a single floor environment but modern buildings are mostly multi-floor.

Compared with the former work in [27], the major technical improvements in the current work can be summarized in three points: (1) Since the model in the conference paper used the Sigmoid as the activation function for Yolo structure, the convergence of the deep neural network required a greatly long iteration. In the new deep neural network, the MobileNet is used for the feature extraction-based SSD structure which allows the deep neural network to quickly converge by only 60,000 iterations; (2) The final layer of the original deep neural network was the fully connected which was computationally very time-consuming, only achieving 13 FPS on the mobile platform. In this paper, we utilize the Depthwise convolutional layers in the SSD instead of the Yolo structure which enables the model to cope with 30 FPS on the mobile platform and retain the same person localization quality; (3) The following robots presented in the conference paper only followed the target in a single floor environment. A new dataset for the situation of the robot operating in the elevator is introduced and an experiment allowing the following robot to move through multiple floors are performed, both of which prove the strength of our robot when operating in multi-floor buildings. 

## 2. Method

Our approach proposes the hypothesis of utilizing an online tracker incorporated with a pre-trained detector, in order to adapt the person-localization model to various environments. In our approach, a deep neural network plays the role of searching for the original position of the user, and restricting the appearance of the target. The part-based tracker localizes the continuous movement of target person’s appearance online. The PN classifier contributes to verifying the tracker’s result, to adapt to environment changes, re-detecting the user if the user is lost. After every tracking step, the model of the tracker and the PN classifier will be updated according to the result in the last frame. Cooperating with the tracker, the PN classifier, and the deep neural network detector, our approach enables a robust person-localization technique with the strength to overcome environment challenges. 

### 2.1. Initialization

**Figure 2:**
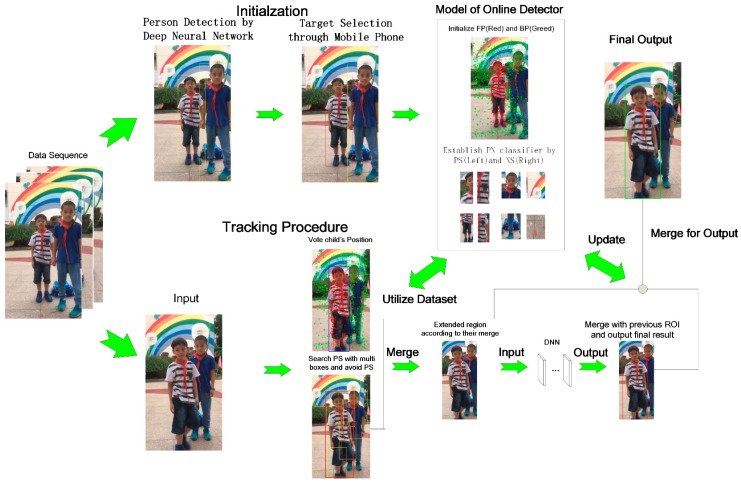
Structure of the person localization model.

The initial position that is input into the tracker is detected by the CNNs neural network described in Section 2.3.2. As shown in Figure 2, our approach begins by initializing the online model (tracker and PN classifier) once the position of the user is detected. A simple human–robot interaction between a mobile phone and a robot system is employed. The mobile phone remotely launches the robot system, and then the robot system begins to localize the person’s position on the scene by a deep neural network, after the user had clicked the start button. Once the user is in the scene, many ROI candidates can be found in the mobile phone’s scene, through the remote desktop on the robot system. The user then only needs to click on his correct position (the correct ROI box on the scene) to start the following task. With such a design, the user can judge the time point to start the following service and avoid incorrect targets if other people are in the scene. According to the ROI output by the detector, our approach divides the region inside the ROI as the foreground (FR), and the outside as the background region (BR). The feature points on the original scene are then detected. There are several valuable feature points that are detected [8,24,28]. In our case, we only need to track the continuous movement of different components on the target, and so the direction estimation of each point was unnecessary. Therefore, our tracker only detects the corner key points on the image, and GFTT [29] was selected because of its efficiency with visual tracking. Binary Robust Invariant Scalable Keypoints (BRISK) [30] were employed as the descriptor for the detected feature points, because the multi-scale-describing strength from the binary description could benefit from the scale challenge during the person-localization procedure. Meanwhile, the positive and negative samples of images were directly defined by the FR and BR. In our approach, we set the properties of the feature points and samples as follows:

(1) $$p,s=\{\begin{array}{c} positive,\;if\;p,s\in \;FR\\ negative,\;if\;p,s\in BR \end{array}$$

where p denotes the feature points detected on the scene, and s means the image samples that will be used to establish a PN classifier. 

Feature points and samples were set to foreground and background points, according to their positions on the scene. For the tracker, every feature point matched all other foreground points. The center of the feature points is voted according to the foreground group cluster. Meanwhile, the rotation and translation among those foreground points are established for the use of the build correspondence of the tracker over continuous frames during tracking. The PN classifier is then established according to the FR and BR. Our approach divides the image into small patches, and saves them as either positive or negative samples, according to their position inside or outside the ROI output of the deep neural network detector. Due to the fact the model is being updated frame-by-frame, we selected the randomized decision forests [31] as the model to describe those images. The randomized decision forests were constructed by many decision trees, and the output it decided by the accumulating accuracy of those trees’ nodes. Due to the imbalance of our training data to the PN classifier (the number of foreground samples is less than that of the background samples), the randomized decision forests could force every decision to be considered by the positive samples, in order to maintain the robustness of the PN classifier.

### 2.2. Tracking

After the initial model of the tracker was established, our approach starts a loop tracking procedure. The image is firstly detected by the GFTT to obtain the feature points’ position. The correspondence between two continuous frames was built up. The Lucas–Kanade optical flow [6], a sparse optical flow method for tracking the same points’ movement between two images, was employed to filter obvious outlier points. A global matching was then employed to build up a more detailed correspondence among the feature points. The function of the global matching is shown as follows:

(2) $$d\left(p_{i}^{t-1},p_{j}^{t}\right)<\theta \wedge \frac{d\left(p_{i}^{t-1},p_{j}^{t}\right)}{d\left(p_{i}^{t-1},p_{k}^{t}\right)}<\gamma ,j\neq k$$

where $p_{i}^{t-1}$ denotes the feature points in the dataset, and $p_{j}^{t}$ are the matched points in the current frame. θ is the threshold of the translations of the matched feature points, and γ is the threshold of the LK optical flow, which are set as 15 pixels and 20 pixels, respectively. The tracker matches the feature points in the current frame with those filtered by the LK optical flow; then brute force matching is utilized to compare each two feature points’ pair, and to build up the correspondence $d\left(p_{i}^{t-1},p_{j}^{t}\right)$. The major reason for using the optical flow to filter the obvious wrong points is to reduce the computing cost of brute forcing. Because the computing cost of brute force is $O\left(i\times i_{i-1}\right)$, the filter by optical flow could significantly improve the speed during processing.

Afterwards, our approach estimates the appearance changes of the object according to the correspondence established by the matching step. The points’ translation is calculated according to the following formula:

(3) $$D\left(m_{i},m_{j}\right)=\|\left(p_{i}^{t}-Hp_{i}^{t-1}\right)-\left(p_{i}^{t}-Hp_{j}^{t-1}\right)\|$$

The Euclidean distance between two matched points $m_{i},m_{j}$ is calculated by a similarity transform H matrix, composed of rotation and scale changes, which are described later. Otherwise, the scale change is measured as follows:

(4) $$s=med\left(\left\{\frac{\left\|p_{i}^{t}-p_{j}^{t}\right\|}{\left\|p_{i}^{t-1}-p_{j}^{t-1}\right\|}\right\},i\neq j\right)$$

The scale ratio s is computed by the median value of the ratio of all pairwise points’ distance changes. Finally, the rotation change was computed according to the following equation:

(5) $$r=\mathrm{med}\left(\left\{arctan\left(p_{i}^{t-1}-p_{j}^{t-1}\right)-arctan\left(p_{i}^{t}-p_{j}^{t}\right),i\neq j\right\}\right)$$

The rotation change r is calculated by the median value of the arctangent changes of all the pairs of the feature points between the two frames.

Once the distance, scale, and rotation changes between two frames are obtained, our approach determines the center of the cluster to obtain the ROI output by the formula:

(6) $$c=\frac{1}{\left|M\right|}\underset{m_{t}\in M}{\sum }\left(p_{i}^{t}-Hp_{i}^{t-1}\right)$$

where *M* denotes the matched points. In function (6), the center of the object is voted based on the average value of all of the center coordinates of the feature points’ pairs. According to this, the oriented position of the ROI is output by simply applying a similarity transform (c,s,r). The tracker finishes the following task, outputting the ROI through the final center voting step. The matching step builds up a relationship between the two frames, i.e., the rotation/scale estimations, to present the appearance change on the object. Depending on them, the final voting outputs a robust and invariant tracking position of the user.

In this part, our approach divided the user’s appearance into various parts, and kept following those parts through their continuous movement. Compared with detecting-by-tracking methods, this step continues to localize onto the user, which is more reasonable, and it adapts the detector to the online environment changes, but this is not limited by the offline status.

### 2.3. Validation and Model Updating

After the tracker calculates the temporary position of the user, the validation step is employed to further improve the robustness of the person’s localization. Our approach divides the validation procedure into two steps: (1) PN classifier checking, and (2) deep learning neural network verification.

#### 2.3.1. PN Classification

Because changes in the user’s appearance and background environment are inevitable during a long-term following task, the adaption for the model to the environment is essential. In our approach, the PN classifier is first used to further check the confidence of the user’s appearance that was output from the tracker. The scene is scanned using different scales and sizes of sliding windows, and then all proposals are input into the PN classifier to output the confidence rates. The selective scan helps the PN classifier rapidly search the user’s potential position globally. The image of each proposal is input into the PN classifier, and the most alike position is output. Random forests plays the role of storing the model of the PN classifier, due to its robustness towards imbalanced data (positive samples are much less abundant than negative samples). The forests are input with structural patches ($x_{i}$) of the given image, and a series of probabilities $p_{r}\left(T|x_{i}\right)=\frac{ps}{ps+ns}$ are output frame-by-frame. The final average value is composed of those probabilities, and our approach selects the window with the best score as the output of the PN classifier. The ROI resulting from the PN classifier is compared with the proposal of the tracker. If the overlapped area is over 80%, our approach selects the tracker’s ROI, and if not, the results of the classifier and the tracker are merged into a new ROI. Our strategy contributes to a smooth ROI change by adequately utilizing the strengths of the tracker and detector, which maintains the stability of the user’s localization.

The PN classifier further improves the robustness of the person localization in this step. Because the tracker in the last step only considers the feature points’ movement, the PN classifier also regards the changed texture information, to in order to have a better evaluation of the person’s position.

#### 2.3.2. Convolutional Neural Network

After the tracker and classifier output the potential ROI of the user on the scene, the deep learning neural network that was trained by our own collected dataset is utilized, to further check the validation of the output. Due to that, our approach aims at operating on a mobile platform without GPU enhancement, and our approach employs depthwise convolution to minimize the computing cost during the CNN and MobileNet as a backnet, to rapidly extract the feature map.

**Figure 3:**
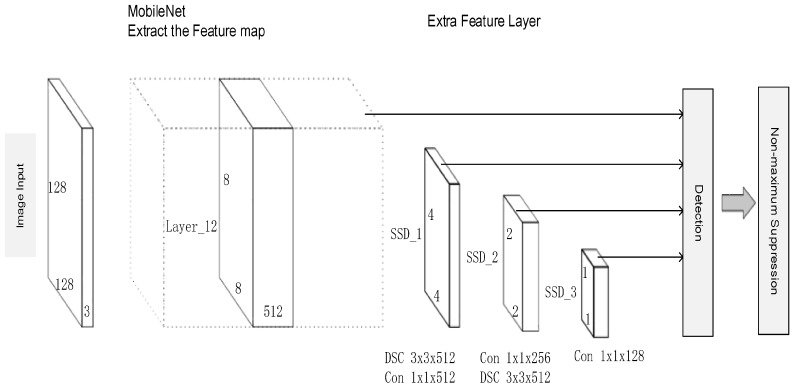
Architecture of the neural network.

The architecture of our proposed neural network is shown in Figure 3. Because the neural network only detects a small region that was extended from the temporary output of the tracker and the PN classifier, we reduced the input of the original SSD from 300 × 300 to 128 × 128, and the number of convolutional layers from five to three, which could sharply reduce the computing cost. The MobileNet is initially employed to extract the input image and to build up the feature map. Three depthwise convolutional layers are then employed to enable our neural network to detect at multi-scales. Finally, non-maximum suppression is employed to select the most confident region to output, and to filter the overlapped ROIs. If the network outputs a score of over 0.8, the result of the proposal from the classifier and the tracker is selected as the final region; otherwise a local search with an extended region of the proposal is employed. Another issue is the situation in which the detected person is overlapped by another person; in the case that two persons are detected on the scene, then the detector chooses the output that is closest to the PN classifier and tracker, because the online learnt features can ideally express the correct person; thus, we only need to simply output the intensity of the whole approach to the online detectors. According to the searching output, the best result is chosen as the ROI, which is merged with the outputs of the tracker and classifier as their median.

The final neural network helps the above two online detectors to restrict the tracking intensity to be the target human, and also to benefits a better ROI output. Different from traditional person-localization approaches, our method incorporates the online learnt features with an offline trained model, which can make our person-localization framework maintain its robustness with high FPS, and adapt to environmental challenges. Compared with the original version described in [27], the current model employs the state-of-art convolutional layer for rapid processing (Depthwise convolutional layers) and adds the basenet using Depthwise convolutional layers (MobileNet) to extract the features better, which benefit both the speed and quality of our model.

#### 2.3.3. Updating

An updating procedure is processed for the purpose of learning environment changes. The updating procedure is similar with that of the initial step, i.e., the image data is divided into foreground and background positions, according to the region that was provided by the final person’s localization result. The difference is that our approach utilizes the feature points of the established model from the tracker, to replace both previous foreground points and background points. For the PN classifier, the region in the foreground group is inserted into the positive samples, and others into the negative samples, which are used to update the PN classifier. In this way, the classifier learns of any environmental change, both in the user’s appearance and the environment background. The tracker only needs to update a limited number of feature points, in order to avoid the issue of accumulating errors.

## 3. Experiments

**Figure 4:**
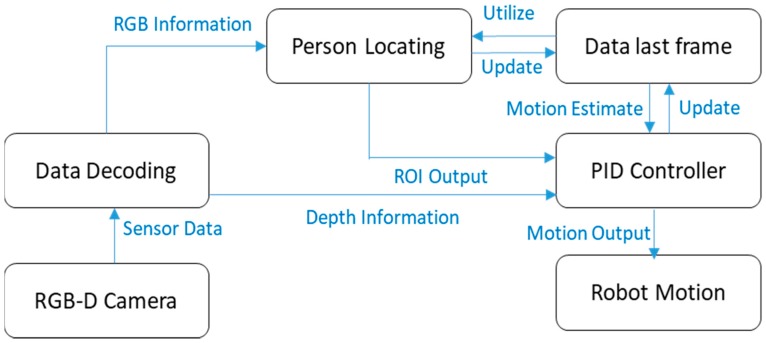
Framework of robot following system.

To evaluate the robustness and performance of our tracking approach, we designed three major scenarios in both static and dynamic scenes, with and without the classification-lock strategy. Figure 4 shows the entire framework for our present robot following system. Our approach was first compared with other state-of-arts methods in three sequences in the Vojir datasets whose targets were humans, so as to prove the advances of our tracking method. This was further tested on static scenes with challenges of feature-rich influences, scale change, appearance change, light influence, and occlusion, to prove the significant improvement of the classification-lock strategy. Afterwards, one dynamic scene was employed to simulate the environment challenges on a real-following task, in order to evaluate the robustness of our approach, during the time in which the robot was moving. Finally, our approach was employed to accomplish following travel in a multi-floor environment.

One issue concerns the narrow space inside an elevator; the solution was to let the user turn around, allowing their front to face the robot, and to let the robot turn back when the elevator arrived at the proposed floor. The mobile robot platform was set up based on a turtle robot, with a simple PID strategy to control the robot to keep a fixed distance of 1.5 m from the user. The real-robot experiment was only operated in an indoor environment, because the optic structure to obtain distance information was influenced by sunlight; thus, outside, the robot would not maintain its distance from the target human, and this would lead to failure of the following experiments. In our experiment, a laptop equipped with an Intel 7700 HQ CPU and 16 GB ddr4 memory was selected to operate our following robot system. To acquire the image and distance information, the prime sense was selected. The prime sense contained a 640 × 480 camera with a widely horizontal angle range and a structured light obtaining precise depth map, which assisted our approach in constantly localization the user on the scene, and obtaining the distance between the user and the robot. During the operation, our approach could achieve about 30 FPS in average during the tracking period with such a mobile platform. 

### 3.1. Training Data

For the purpose of training the CNNs detector, an in-house dataset was collected (1255 images) with two classes, upper body, and whole body. In our dataset, Shenlu Jiang, Ling Li and Cheng Su played as the models for some of datasets and 60% datasets are collected from other colleagues with consent to use them in the dataset. The other 40% of the dataset is collected from the Internet with legal license. The modeling of the upper body was employed for the following task on real-world experiments. Because our robot set a fixed distance of 1.5 m between it and its target human, the scale of the human was too large, and the camera could not capture the entire appearance of the human at this distance; thus, we employed the upper body detector to localize the human. The whole-body model was utilized for the purpose of evaluating our model in the sequences of static views and the Vojir dataset.

**Figure 5:**
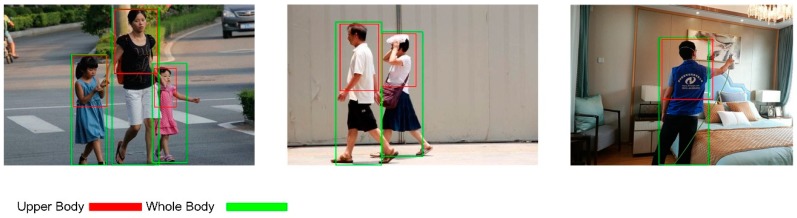
Sample images for training.

One factor that should be noted is that the whole and upper body datasets were trained and tested individually, but not when mixed together. As shown in Figure 5, our own designed dataset included various rotations, different poses, and different scale of humans, which confirmed the robustness of the detector to localize the person. We then divided 400 images as test images to evaluate the performance of the neural network (in the followed experiments, all data was be used to train the model). The mean average precision (MaP) is used in the evaluation.

**Figure 6:**
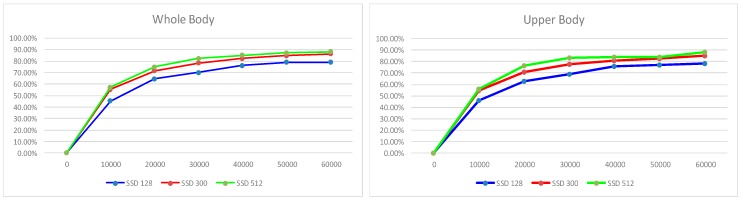
Training procedure of whole body and upper body, accuracy vs iterations.

As shown in Figure 6, the SSD with a 512 × 512 input resolution obtained higher accuracies than the other two in whole- and upper-body tests (89.1% and 88.5%). We tested SSD 300 and 512 in our platform, and calculated their own perspective processing times. SSD 300 took approximately 130 ms to process each image, and SSD 512 took approximately 300 ms to process each image. Compared with SSD 128 (about 60 ms), although the mode in the other two resolutions obtained higher accuracies, the high computing costs prevented them from operating in our robot system on a mobile platform in real-time. Due to the fact that the neural network only detected the user’s position in a slightly extended region after the tracker and PN classifiers, the SSD 128 (70.1% and 69.8%) was sufficient to fulfil the person-localization task. One other task for SSD 128 was to provide the potential positions of the user on the scene during the initialization step. The SSD 128 only achieved a 69.8% map, and this approach mainly failed in a crowded environment, because the image resolution of the test dataset was much higher than 128 × 128. Thus, we designed a simple initializing environment, i.e., a single person standing alone on the scene and using a mobile phone to remotely connect the robot to get the scene on a robot operating a PC. The user interacted with the robot by simply clicking the region of the target person in order to select the original region of the user on the robot scene.

### 3.2. Benchmark Comparison

**Figure 7:**
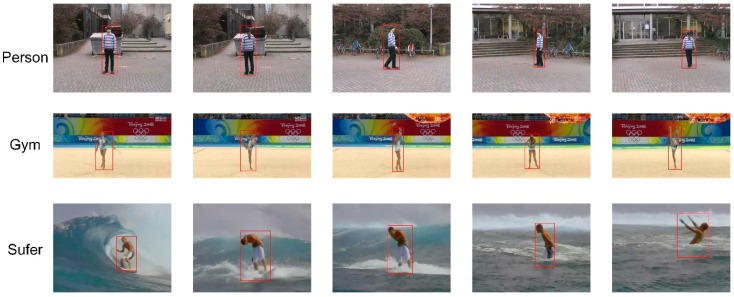
Image sequences for the Vojir dataset.

First of all, our tracking approach was compared with other state-of-the-art approaches on a common dataset to prove the advance of our tracking quality on a human target. There were three sequences: gym, surfer, and person, which were selected from the Vojir tracking dataset (available online via the link in [26], as shown in Figure 7). The target objects of the three sequences were humans, which included scale, appearance, and rotation changes:

(7) $$R=\frac{TP}{TP+FN}$$

The recall rate was used to evaluate the tracking performance. Its formula is shown in Equation (7). TP is a true positive object, and FN is a false negative object. In the evaluation, we regarded the outputs with overlapped regions with the ground truth ROI ≥ 0.8 being the true positive, and less than 0.8 being the false positive. Comparing with simply using precision to evaluate the models, the recall rate sufficiently evaluated the situation of localization the failure, and it could obtain a more statistical result. The recall rate of each item is calculated in Table 1. The state-of-art methods of tracking, STR [17], CT [32], STC [33] TLD [16], and CMT [18] were used to compare with our approach.

According to the results shown on Table 1, STR received the two best scores in the gym and person sequences, because a great number of appearance changes existed in the two scenes, which proved the success of tracking-by-detecting with learner updating. However, a recall rate of 0.46 in the surfer sequence showed the risk of STR during a great scale change. STR’s similar approach of TLD received a median score in all items, since the simple modeling method restricted its abilities in updating the model and the tracking object.

The whole appearance tracker CT received the same score as STR in the gym sequence, but it failed in the surfer and person sequences. Such a result shows that the whole appearance tracker did not have the ability to overcome scale changes, but it did well for appearance changes. STC also received a poor score, because the scale change issue was similar to CT. The part-based tracker CMT received good scores for all three items; however, the static geometric structure caused a limitation for CMT, with significant changes occurring in the surfer and person sequences. Our approach without a classification-lock was the second-best recall rate on average, and it proved that our strategy of tracking could overcome common challenges in visual tracking. Last but not least, the classification-lock strategy improved our approach with higher precision, which allowed our approach to achieve the best score on average. However, our approach received a lower score in the surfer sequence than without a classification-lock. This was due to a part-based influence, i.e., we input some part-based situations into our dataset that allowed the classification to estimate the person’s appearance, even if part of the body was covered. The surfer benchmark labeled the coordinates of the person’s upper body after partial occlusion occurred, and this was the main reason for our score being lower than that without a classification-lock. Apart from this, our approach performances were 32.7, 28.9, and 30.1 FPS in the person, gym, and surfer sequences, respectively (because the number of feature points on a person’s appearance was different), which was sufficient to fulfill the processing on the following robot.

The results proved the efficiency and advance of our tracking approach, which achieved significantly higher accuracies than other state-of-art methods. Comparing with original version, the FPS of our model triple increase from 13 FPS to 30 (in average) on a mobile platform, and it keeps the same level accuracy in the visual tracking benchmark comparison.

**Figure 8:**
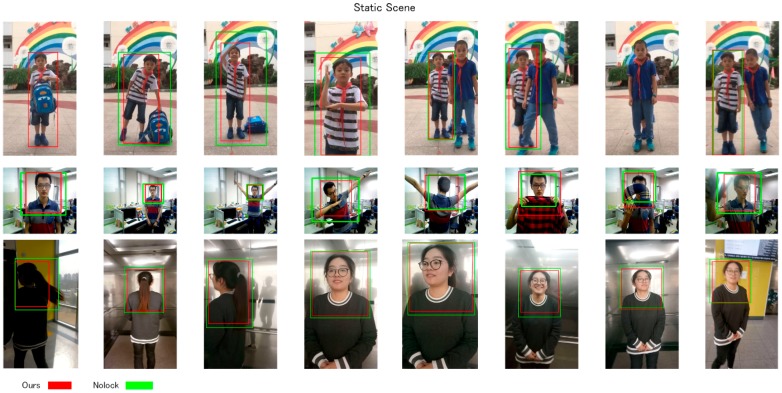
Image sequences for the static view.

Our approach then further evaluated the efficiency of a classification-lock strategy in a person-following task. Because the two sequences and the dynamic were evaluated as real robot simulations, the input ROI was detected by SSD128, which was different from visual tracking (human sets). The ROI detected by the SSD was not absolutely solid, but the visual tracking methods needed a stable coordinate of ROI to input to the tracker in the first frame; thus, the state-of-art trackers were not compared with our approaches in the dynamic and static scenes. Since various kind of challenges with visual tracking may happen at the same time, we employed various poses, with those challenges being acted on by the user in the three image sequences, and the results are shown in Figure 8 and Table 2. Our approach without a classification-lock strategy was evaluated.

### 3.3. Static Scene

In the first sequence, a child conducted a series of movements on a feature-rich school bag with his hand, to show the influence of the feature-rich inner object’s influence. He then formed several poses with different scales on the scene, frequently accompanied by user walking. Finally, an occlusion by his classmate occurred, in order to simulate an overlapping situation with the target person. In this sequence, a feature-rich influence, scale changes, appearance changes, and occlusion were included for the purpose of evaluating the adaptation of our tracking strategy with those challenges. According to the results, our approach with the classification-lock achieved a 0.96 recall rate, and localized the correct child with suitable ROI during the whole period of this sequence. However, the approach without a classification-lock failed in the feature-rich influence risk, because the feature points were mainly distributed on the bag, which hindered the tracker from maintaining localization of the child. The classification-lock strategy contributed in this part, which pulled back the ROI when the tracker proposed to localize the error target. Therefore, we proved the accuracy of our approach in the above challenges, and showed the efficiency of our approach to localize the user against scale and appearance change influences, even when they occurred together.

In the second sequence, the developer performed a series of rotation behaviors in different scales, which consisted of sudden scale, rotation, and appearance changes. Both our approaches with and without classification maintained constant tracking of the object. However, the implementations with ROI and with the classification-lock acted more stable than the ones without these properties, during the whole tracking procedure. The major reason was that the unstable feature points during the scale and appearance changed. During the movement of the target, the distribution of the feature points greatly moved, and this may have caused the occurrence of a temperately unstable ROI for the target person. In such case, the classification-lock strategy assisted the tracker to localize the person better, with a 0.08% higher recall rate. In this sequence, our approach was illustrated to be able to overcome rotation change in different scales, overlaps, and feature-rich challenges. 

In the third sequence, the developer walked into an elevator, rotated inside the evaluator, using the front face to the follower, and walked out when the elevator door opened. This sequence consisted of an exposure and a darkness situation, which simulated the situation of our robot moving among the floors. Both of our approaches with and without a classification-lock strategy could keep constant tracking intensity on the target human, but the one with a classification-lock strategy showed more a precise ROI output during the point at which the darkness caused an unstable trend and decrease in the number of feature points.

The recall rates of our approach were also higher than without a classification lock strategy. The three sequences illustrated that our classification-lock strategy was able to further improve the ROI output of the tracker, and to avoid the occurrence of the accumulation error on the tracker. Furthermore, the classification-lock strategy also helped the tracker to work against failure when the feature points were unstable. In additional, our approach managed to efficiently overcome scale and appearance changes with the lock present, or not (except for the feature-rich object influence) in these static scenes.

### 3.4. Dynamic Scene

**Figure 9:**
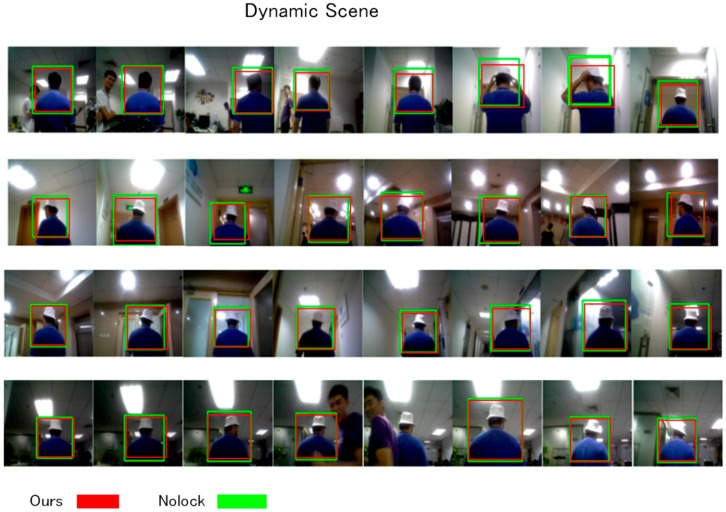
Image sequence for the dynamic situation.

In the dynamic scene, a designed task was employed to simulate a real case that could happen to the person being followed. This image sequence was recorded on a rainy day inside our office, when the illumination situation was awful. In this experimental image sequence, our office served as the background, which had complicated light situations and numerous background features. We designed a series of challenges described in the dynamic view, and the image sequence in Figure 9 shows this travel, in which the background environment was complicated enough to evaluate the robustness of our approach.

Our robot initialized the user’s model at an initial position, and then it began to follow the user in the office environment. During this travel, our person-localization approach worked against the influence of a complex background and the illumination changes in different rooms. The robot determined the appearance changes of the user when he decided to wear a hat. Occlusions occurred three times during the following task, and our approach rapidly re-detected the user correctly. We also allowed the user to speed up and periodically pause during the experiment, which made large-scale changes to the scene, and our robot successfully continued to follow the user, even when their scale changed greatly.

**Figure 10:**
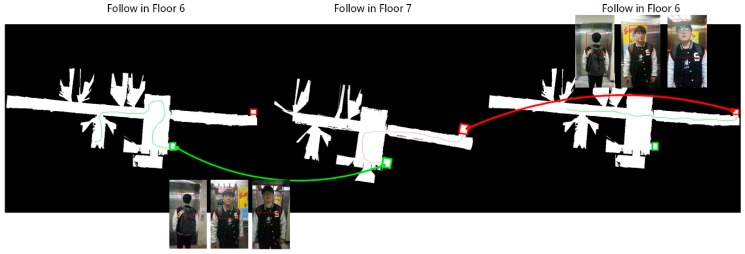
Path during a multi-floor following experiment.

Our approach was then operated as a real-world following task in a multi-floor environment. One factor was the human–robot interaction. Our system employed a simple APP in a cell phone to communicate with the robot remotely. During the initialization, the user could watch the APP to check the detector’s result in real-time, and select his position to begin following. During the operation, the APP could allow the user to stop and re-start the following function. As shown in Figure 10, our robot first began its travel in our room. The user then turned around in the lobby and entered an elevator. Inside the elevator, the user turned make their front face towards the robot. Once the elevator arrived at the correct floor, the user went straight ahead to make the robot turn back to move outside the elevator. Afterwards, the user roamed on the fifth floor, and finally moved to another elevator. The behavior of the user in the elevator was the same as when he controlled the first elevator back to the seventh floor. Finally, the user returned to the original place and finished their travel. 

The result shows that our approach on a single floor constantly localized the correct target against appearance changes, scale changes, and illumination challenges, even though some scenes were extremely dark or exposed. The classification-lock strategy collaborated with the trackers, which further improved the tracking quality. The classification-lock strategy also made the ROI output more reasonable, based on previous perceptions of human appearance, and this benefitted the tracker when the tracker lost feature points in the scene. The successful travel over multiple floors illustrates that our approach had the strength to be a robust follower and serve in the office environment, even while using a simple PID controller. 

## 4. Conclusions

This paper illustrates an adaptive tracking approach where the CNNs detector is incorporated with the tracker by using a classification-lock strategy, and it is applied to a mobile platform. Our approach obtains a 91% recall rate, and an 87% recall rate without a classification-lock strategy in the benchmark comparison, which are both 10% higher than the method that was ranked second. The results illustrate the efficiency of our tracker in common challenges during the human following task. In the evaluation of the static view, our approach achieves an approximately 8% higher recall rate than that without a classification-lock strategy on average, which proves that the classification-lock strategy can benefit from the performance of person localization. In a real-world experiment, the robot system deployed by our person-localization approach obtains precise results, which proves the robustness of our person-localization approach in a dynamic scene with specifically designed challenges. Moreover, our approach can also successfully operate in a complicated multi-floor indoor building. In summary, our approach builds a bridge between practical human perception and traditional visual tracking methods, to greatly improve and accomplish person-following tasks. 

## Figures and Tables

**Table 1:** Recall rate comparison with state-of-art methods of tracking.

Sequence	Methods						
	STR	CT	STC	TLD	CMT	Unlock	Ours
Person	0.80	0.01	0.26	0.60	0.57	0.71	0.79
Gym	1.00	1.00	0.57	0.50	0.80	0.94	0.98
Surfer	0.46	0.01	0.24	0.36	0.43	0.97	0.96
Average	0.75	0.33	0.35	0.48	0.60	0.87	0.91

**Table 2:** Comparison of our approach with and without classification-lock strategy.

Methods	Sequences		
	Child	Man	Woman
Ours	0.96	0.95	0.98
No lock	0.85	0.87	0.96
